# Triglyceride glucose index and poor sleep patterns in non-diabetic adults: Evidence from NHANES 2005–2016

**DOI:** 10.3389/fnut.2023.1051667

**Published:** 2023-01-30

**Authors:** Chi-Feng Liu, Li-Wei Chien

**Affiliations:** ^1^School of Nursing, National Taipei University of Nursing and Health Science, Taipei, Taiwan; ^2^Department of Obstetrics and Gynecology, School of Medicine, College of Medicine, Taipei Medical University, Taipei, Taiwan; ^3^Department of Obstetrics and Gynecology, Taipei Medical University Hospital, Taipei, Taiwan

**Keywords:** sleep, triglyceride-glucose index (TyG), insulin resistance (IR), National Health and Nutrition Examination Survey (NHANES), patterns

## Abstract

**Introduction:**

Sleep disorders are commonly encountered in modern populations. This cross-sectional study aimed to investigate the associations between triglyceride glucose (TyG) index and poor sleep patterns in non-diabetic adults.

**Methods:**

Data of non-diabetic adults aged 20–70 years were extracted from the US National Health and Nutrition Examination Survey database 2005–2016. Pregnant women, individuals with diabetes and cancer history, and individuals lacking complete data on sleep patterns or parameters for calculating TyG index were excluded. Poor sleep pattern was defined as having two or more following conditions: (1) abnormal sleep duration, defined as less than 7 h or longer than 9 h; (2) self-reported trouble sleeping; and (3) physician-confirmed sleep disorders. Associations between poor sleep patterns, TyG index, and an additional index incorporating body mass index (BMI), TyGBMI, and other study variables were determined by univariable and multivariable logistic regression analysis.

**Results:**

Among 9,390 included participants, 1,422 had poor sleep patterns and 7,968 did not. The individuals with poor sleep patterns had a higher mean TyG index, were older, had higher BMI, and had higher proportions of hypertension and history of CVD than those without poor sleep pattern (all *p* < 0.001). Multivariable analysis showed no significant association between poor sleep pattern and TyG index. However, among the components of poor sleep pattern, TyG index in the highest quartile (Q4) was significantly associated with trouble sleeping [adjusted OR (aOR): 1.46, 95%CI: 1.04–2.03) as compared with the lowest TyG quartile (Q1). In addition, TyG-BMI in Q4 was indepently associated with increased likelihood for poor sleep patterns (aOR: 2.18, 95%CI: 1.61–2.95), trouble sleeping (aOR: 1.76, 95%CI: 1.30–2.39), abnormal sleep duration (aOR: 1.41, 95%CI: 1.12–1.78), and sleep disorders (aOR: 3.11, 95%CI: 2.08–4.64) as compared to Q1.

**Discussion:**

Among US adults without diabetes, elevated TyG index is correlated with self-reported trouble sleeping, independent of BMI. Future studies should build upon this preliminary work and examine these associations longitudinally and through treatment trials.

## 1. Introduction

Insulin resistance (IR) is defined clinically as the inability of a known quantity of exogenous or endogenous insulin to increase glucose uptake and utilization in an individual to an extent corresponding to that in the average population (1). A state of insensitivity is found in the peripheral tissues in IR, which is recognized as a significant pathological feature of type 2 diabetes mellitus (T2DM). IR indicates impaired glucose uptake, reduction in glycogenesis synthesis, and decremental suppression of lipid oxidation. Insulin secretion increases in the presence of such conditions to maintain glucose homeostasis, leading further to chronic hyperinsulinemia and subsequent elevated oxidative stress and inflammatory responses (2). IR may also impair lipid and serum uric acid metabolism and is thus associated with metabolic syndrome, for which quick and accurate quantification of IR is essential in clinical practice (3).

The triglyceride-glucose (TyG) index, a product derived from fasting levels of triglycerides and glucose is calculated as follows: ln [triglyceride (mg/dL) ×)iglyceride (mg/dL)) fdL)/2]. Given its relevance to lipids and glucotoxicity, TyG index has recently been suggested as a surrogate marker for metabolic dysregulation and IR (4, 5). Specifically, IR, being a metabolic dysregulation, is linked to dyslipidemia. Lipotoxicity not only induces IR, but also impairs β cell function especially in high-glucose environment (6, 7). Further, TyG index does not need an insulin assay thus is less costly than other estimates for IR such as homeostasis model assessment-estimated insulin resistance (HOMA-IR), thus could have wider applications (8). According to the medical literature, TyG index is closely associated with cardiometabolic conditions in the general population, including diabetes, arterial stiffness, hypertension, cardiovascular disease (CVD), and stroke (9, 10).

The important role of sleep in maintaining health and wellbeing has become increasingly recognized. Insufficient sleep and sleep-related disorders markedly affect individuals’ normal daily function and quality of life (11). Among the US adults aged 20–79 years, 16.3% suffered from sleep issues (12). The diagnosis and treatment of poor sleep and sleep disorders are of common interest to multiple medical disciplines. Recent studies have proposed that irregular sleep duration is a risk factor for CVD, cardiometabolic disease, hypertension, stroke, and arterial stiffness (13–17). On the other hand, a growing body of research shows that inadequate and poor-quality sleep is associated with changes in insulin resistance and β cell function, potentially leading to metabolic dysregulation and subsequent type 2 diabetes (18, 19).

It is essential to detect alterations of individuals’ metabolic status and its potential association with poor sleep pattern to prevent type 2 diabetes. Beyond the conventional IR-related measures, the relationships between sleep-related conditions and the TyG index have yet to be explored. Therefore, this study aimed to evaluate the potential association between individuals’ poor sleep patterns, TyG index, and a closely related index incorporating BMI, TyG-BMI, in non-diabetic adults using a nationally representative database of the US.

## 2. Materials and methods

### 2.1. Data source

This cross-sectional study analyzed secondary data extracted from The National Health and Nutrition Examination Survey (NHANES) database. NHANES is a program of the National Center for Health Statistics (NCHS), which is part of the Centers for Disease Control and Prevention (CDC) in the USA.^1^ The ongoing series of surveys combines interviews and examinations to evaluate the health and nutritional status of adults and children. It uses a complex, multistage design to collect and analyze data representative of the national, non-institutionalized population of the USA. The data are released for research purposes, and permission to use the data is granted to researchers by the NCHS. Participants in NHANES complete a household interview and are invited for an extensive examination in a mobile examination center (MEC), including a physical exam, specialized measurements, and laboratory tests. Consequently, evaluating subjects within the NHANES database is reliable and multidimensional and is equated with a population-level assessment (20).

### 2.2. Ethical considerations

The NCHS Research Ethics Review Board reviewed and approved the NHANES program, and all survey participants provided signed informed consent. Therefore, no further ethical approval or informed consent was required to perform the analysis of secondary data reported in this manuscript. Please check the NHANES website for NCHS Research Ethics Review Board Approval.^2^ Additionally, all NHANES data released by the NCHS are de-identified, and the data remain anonymous during data analysis.

### 2.3. Study population

Participants’ data were extracted from released cycles of the NHANES database 2005–2016. The inclusion criteria were non-diabetic adults aged 20–79 years. Exclusion criteria were: pregnant women, individuals with a history of diabetes or cancer, and individuals lacking complete data on components to define poor sleep patterns or parameters for calculating TyG. Non-diabetic population were focused for two reasons: (1) diabetes patients could be on lipid-lowering medications concurrently, thereby TyG index could be confounded; (2) we would like to capture the link between metabolic change and sleep BEFORE diabetes occurs, such that to assist in the development of timely strategies to stop the deterioration from early mild metabolic dysregulation to diabetes. Participants with a cancer history were excluded, for it is well known that tumor cells grab most of the nutrition and energy which may influence glucose and lipid levels, and there is a known strong relationship between cancers and sleep problems that could mask the association of interest. Participants with diabetes were identified through at least one of the following questions and were excluded from the study cohort: a positive response to the questionnaire “Are you taking insulin?” “Did a doctor tell you that you have diabetes?” “Do you take pills to lower blood sugar?” or an HbA1c ≥ 6.5%, fasting glucose ≥ 126 mg/dl, or a glucose level ≥200 mg/dl in oral glucose tolerance test (OGTT) as recorded in the NHANES-derived laboratory data (21).

### 2.4. Study variables

#### 2.4.1. Assessment of sleep factors and definition of poor sleep pattern

Participants’ nighttime sleep hours were obtained by responding to “How much sleep do you usually get at night on weekdays or workdays?”. The sleep duration was categorized as abnormal (<7 h or >9 h per night) and average (7–9 h per night). We defined short sleep as sleep duration <7 h according to the consensus recommendation by the American Academy of Sleep Medicine and the Sleep Research Society for the amount of sleep needed to (22). The response to “Have you ever told a doctor or other health professional that you have trouble sleeping?” was used to assess self-reported trouble sleeping. The answer to “Have you ever been told by a doctor or other health professional that you have a sleep disorder?” was used to assess the presence of sleep disorder or not., we defined “poor sleep patterns,” a composite sleep measure used in a previous NHANES study, as the presence of two or more abnormal sleep factors, including variations in sleep duration (<7 h or >9 h), trouble sleeping, and physician-confirmed sleep disorders (23).

#### 2.4.2. Measurement of TyG and tyG-bMI

Blood specimen collection was conducted in the morning after at least nine hours of overnight fasting. The hexokinase method was used to measure fasting blood glucose. The measurement of serum triglycerides (TG) was performed using enzymatic assays. TyG index was calculated using the following formula: TyG = Ln[fasting triglycerides (mg/dl) × fasting glucose (mg/dl)/2] (24).

Since a recent study documented that TyG-BMI was superior to TyG for IR prediction (25), we also included TyG-BMI, calculated as follows, for additional analyses. TyG-BMI = Ln [fasting triglycerides (mg/dl) × fasting glucose (mg/dl)/2] × BMI. The TyG and TyG-BMI were also divided into quartiles for the analysis of the association with poor sleep patten. Of note, there is currently no consensus on ‘normal value’ of TyG or TyG-BMI. Nevertheless, in a previous NHANES study, among the US general population over 40 years of age, TyG ranged from 6.79 to 12.55 with a mean of 8.8 (26).

### 2.5. Covariates

Demographic data, including age, gender, race, and income-to-poverty ratio, were obtained through in-person interviews conducted by trained interviewers using the Family and Sample Person Demographics questionnaires and the Computer-Assisted Personal Interviewing (CAPI) system (Confirmit Corp. New York, USA). Collected data were weighted according to the NHANES protocol.

The body mass index (BMI) value was obtained from NHANES examination measurements, calculated as body weight (kg) divided by height (m^2^). Body weight was measured using an electronic load cell scale, and standing height was measured using a fixed stadiometer.

Hypertension was defined as responding “yes” to the questions: “Were you told on two or more different visits that you had hypertension, also called high blood pressure?” or “Because of your (high blood pressure/hypertension), have you ever been told to take prescribed medicine?”, or with an average of three consecutive measures on systolic blood pressure ≥140 mmHg, or with an average of three successive measurements on diastolic blood pressure ≥90 mmHg.

Cardiovascular disease (CVD), including coronary heart disease, angina, congestive heart failure, myocardial infarction, and stroke, were defined by the question: “Has a doctor or other health professional ever told you that you have (disease)”.

Glomerular filtration rate (GFR) was estimated from re-calibrated serum creatinine using the 4-variable Modification of Diet in Renal Disease (MDRD) Study equation. Here we used the IDMS-traceable MDRD Study equation that uses standardized creatinine: GFR = 175 × (standardized serum creatinine) − 1.154 × (age) − 0.203 × 0.742 (for female participants) × 1.212 (for black participants). Estimated GFR is reported in ml/min/1.73 m^2^. Chronic kidney disease (CKD) was defined by an eGFR <60 ml/min/1.73 m^2^.

Smoking status of participants was classified as non-smoker, former smoker, or current smoker, defined as follows: lifetime smoking of fewer than 100 cigarettes was a non-smoker; lifetime smoking >100 cigarettes but not currently a smoker was a former smoker; lifetime smoking >100 cigarettes and responding “yes” to the question: “Do you smoke now?” was a current smoker.

Alcohol consumption was classified according to responses to the survey questions defining alcohol consumption. Excessive alcohol consumption was defined by responses of ≥4 times/week to the question: “In the past 12 months, how often did you drink any alcoholic beverage?”.

### 2.6. Statistical analysis

In consideration of the complex sampling design for NHANES data, all analyses were performed using SAS survey analysis statements to generate nationally representative estimates (SAS Institute Inc., Cary, NC, USA). Weighted samples (WTSAF2YR), stratum (SDMVSTRA), and cluster (SDMVPSU) were used to represent the nationwide analyses. Continuous variables are presented in weighted mean values and standard error; categorical variables are presented in unweighted numbers and weighted proportions. Since the analysis combined data from six survey cycles (2005–2016), sample weights across the survey cycles were constructed according to analytic guidelines published by the National Center for Health Statistics. Differences in means between the subject groups with or without poor sleep patterns were compared using the SURVEYREG statement for continuous variables. The Rao-Scott chi-square test was also performed to determine differences in the proportions between the groups using the SURVEYFREQ statement for categorical variables. Logistic regression was performed using the SURVEYLOGISTIC statement to assess the associations between poor sleep patterns and TyG index or TyG-BMI. TyG and TyG-BMI were used both as continuous variables and as categorical variables in separate models. Significant covariates from the univariable analysis were adjusted for in the multivariable models. A two-sided *p*-value of <0.05 was regarded as statistically significant.

## 3. Results

### 3.1. Study selection

**FIGURE 1:**
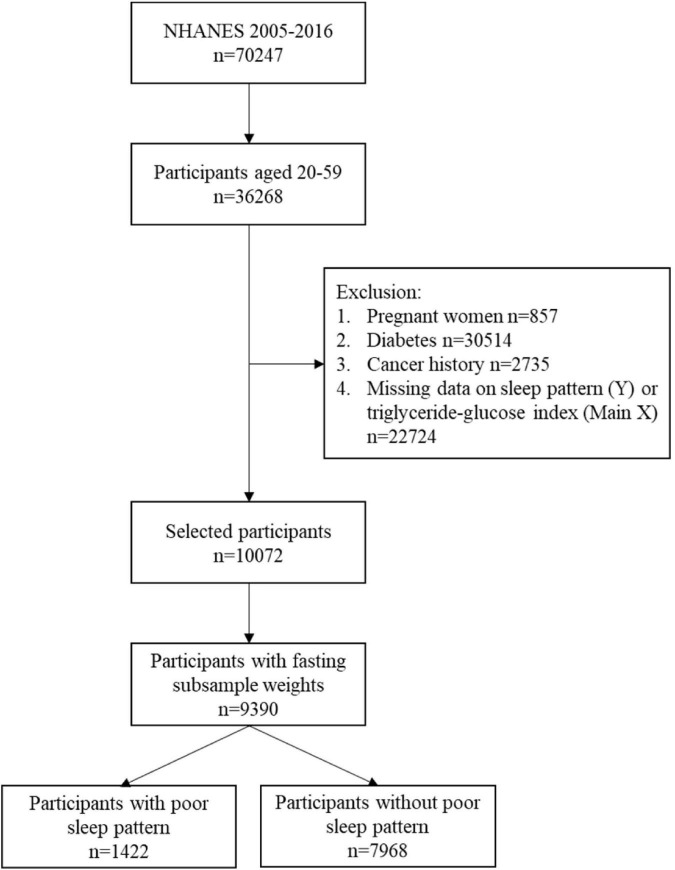
Flow diagram of study population selection.

A total of 70,247 subjects were identified in the 2005–2006, 2007–2008, 2009–2010, 2011–2012, 2013–2014, and 2015–2016 cycles of NHANES. Of these, 36,268 participants aged 20–79 years were included, but only 46.5% (*n* = 16,852) participants were subsampled to fast before attending a NHANES morning MEC exam session. After excluding missing value on TyG index and sleep pattern, remained 13,544 participants having complete data. Further excluding pregnant women, participants with diabetes, or cancer history, 10,072 participants were included in the study cohort. The participant data without fasting subsample weights were further excluded. Finally, 9,390 participants, presenting 169,911,886 people of the US population, consisting of 1,422 individuals with poor sleep patterns, and 7,968 participants without poor sleep patterns were included in the analysis. The flow chart of study inclusion and exclusion is presented in Figure 1.

### 3.2. Characteristics of the non-diabetic adults

The mean age of the study cohort was 41.8 years. Most participants were non-Hispanic White (69.7%), income-to-poverty ratio >1 (87.3%), and were never smokers (55.2%). The mean TyG index was 8.5. Compared with participants with poor sleep patterns, participants without poor sleep patterns had a significantly lower mean TyG index, younger age, and lower BMI. Regarding comorbidities, 23.4% of the study cohort had hypertension (Table 1). The group with poor sleep patterns had a significantly higher proportion of hypertension and CVD history than those without poor sleep patterns.

**TABLE 1:** Characteristics of study population.

Study variables	Overall *N* = 169,911,886 *n* = 9,390	Poor sleep patterns		*P*-value
		Yes (*n* = 1,422)	No (*n* = 7,968)	
**TyG index**	8.5 ± 0.01	8.6 ± 0.03	8.5 ± 0.01	**<0.001**
Q1 (<8.16)	2,708 (29.3)	332 (22.6)	2,376 (30.4)	**<0.001**
Q2 (≥8.16, <8.58)	2,620 (27.6)	379 (27.8)	2,241 (27.6)	
Q3 (≥8.58, <9.03)	2,416 (25.3)	388 (25.6)	2,028 (25.3)	
Q4 (≥9.03)	1,646 (17.8)	323 (24.0)	1,323 (16.7)	
**Age, years**	41.8 ± 0.3	44.7 ± 0.6	41.4 ± 0.4	**<0.001**
20–29	2,208 (25.4)	220 (18.1)	1,988 (26.6)	**<0.001**
30–39	2,021 (21.7)	247 (18.7)	1,774 (22.2)	
40–49	1,950 (22.4)	333 (25.6)	1,617 (21.9)	
50–59	1,490 (17.5)	314 (22.4)	1,176 (16.7)	
60–69	1,105 (8.8)	189 (9.6)	916 (8.6)	
70–79	616 (4.3)	119 (5.6)	497 (4.1)	
**Gender**				
Male	4,702 (50.5)	649 (48.3)	4,053 (50.9)	0.428
Female	4,688 (49.5)	773 (51.7)	3,915 (49.1)	
**Race**				
Non-Hispanic White	4,275 (69.7)	726 (72.7)	3,549 (69.3)	**<0.001**
Non-Hispanic Black	1,907 (10.9)	341 (13.7)	1,566 (10.5)	
Hispanic	846 (5.1)	123 (4.1)	723 (5.3)	
Others	2,362 (14.2)	232 (9.6)	2,130 (15.0)	
**BMI (kg/m^2^)**	28.0 ± 0.1	30.0 ± 0.4	27.2 ± 0.1	**<0.001**
Underweight (<18.5)	179 (1.9)	18 (1.2)	161 (2.1)	**<0.001**
Normal (18.5–24.9)	2,967 (33.7)	330 (25.4)	2,637 (35.1)	
Overweight (25–29.9)	3,145 (33.4)	434 (30.7)	2,711 (33.9)	
Obesity (≥30)	3,030 (31.0)	621 (42.7)	2,409 (29.0)	
Missing	69	19	50	
**Income-to-poverty ratio**				
>1	7,059 (87.3)	1,010 (83.2)	6,049 (88.0)	**<0.001**
≤1	1,730 (12.7)	334 (16.8)	1,396 (12.0)	
Missing	601	78	523	
**Smoking status**				
Never	5,308 (55.2)	664 (45.3)	4,644 (56.9)	**<0.001**
Former	1,958 (22.0)	287 (20.9)	1,671 (22.2)	
Current	2,119 (22.7)	471 (33.9)	1,648 (20.9)	
Missing	5	0	5	
Excessive alcohol consumption	678 (8.9)	77 (7.6)	601 (9.1)	0.484
Hypertension	2,484 (23.4)	599 (35.9)	1,885 (21.2)	**<0.001**
CVD history	476 (4.4)	176 (10.6)	300 (3.3)	**<0.001**
CKD	415 (4.1)	85 (5.6)	330 (3.9)	0.078

BMI, body mass index; CVD, cardiovascular disease; CKD, chronic kidney disease; TyG index, triglyceride-glucose index.

Significant results (*p* < 0.05) were shown in bold.

### 3.3. Associations between TyG index and poor sleep patterns

In the univariable analysis, an increase in the TyG index significantly correlated with increased odds of the presence of poor sleep patterns, trouble sleeping, and sleep disorders (Table 2). The odds of poor sleep pattern, trouble sleeping, and sleep disorder increased with higher TyG quartiles. After adjusting for confounders in the multivariable analysis, the highest quartile (Q4) of the TyG index was significantly associated with increased odds of trouble sleeping as compared with the lowest quartile (Q1) (aOR: 1.46, 95%CI:1. 04–2.03), whereas no significant associations were observed between poor sleep, abnormal sleep duration, sleep disorder and TyG.

**TABLE 2:** Associations between TyG index, poor sleep patterns and its components.

	TyG index			
	Continuous	Categorical: ref: Q1 (<8.16)		
	Per 1 unit	Q2 (≥8.16, <8.58)	Q3 (≥8.58, <9.03)	Q4 (≥9.03)
**Univariate, OR (95%CI)**				
Poor sleep patterns	**1.38 (1.17–1.62)**	**1.35 (1.03–1.77)**	**1.37 (1.10–1.70)**	**1.93 (1.37–2.74)**
Abnormal sleep duration	1.12 (0.998–1.26)	1.20 (0.95–1.51)	**1.28 (1.08–1.52)**	1.15 (0.94–1.40)
Trouble sleeping	**1.35 (1.15–1.58)**	1.21 (0.98–1.48)	**1.47 (1.15–1.87)**	**1.81 (1.35–2.44)**
Sleep disorders	**1.30 (1.03–1.64)**	1.26 (0.91–1.75)	1.34 (0.93–1.95)	**1.81 (1.18–2.78)**
**Multivariable, aOR (95%CI)**				
Poor sleep patterns^a^	1.07 (0.84–1.35)	1.08 (0.79–1.47)	0.96 (0.71–1.31)	1.23 (0.79–1.93)
Abnormal sleep duration^b^	1.01 (0.87–1.18)	1.14 (0.90–1.44)	1.18 (0.97–1.45)	0.94 (0.71–1.23)
Trouble sleeping^c^	1.18 (0.98–1.43)	1.08 (0.88–1.32)	1.28 (0.99–1.65)	**1.46 (1.04–2.03)**
Sleep disorders^d^	0.88 (0.68–1.14)	0.95 (0.69–1.31)	0.82 (0.53–1.25)	0.93 (0.60–1.46)

^a^Adjusted for age, race, BMI, poverty income ratio, smoking status, hypertension, and CVD history.

^b^Adjusted for age, sex, race, BMI, poverty income ratio, smoking status, excessive alcohol con-sumption, hypertension, and CVD history.

^c^Adjusted for age, sex, race, BMI, smoking status, hypertension, CVD, and CKD history.

^d^Adjusted for age, BMI, poverty income ratio, smoking status, excessive alcohol consumption, hypertension, and CVD history.

BMI, body mass index; CVD, cardiovascular disease; CKD, chronic kidney disease; TyG, triglyceride-glucose index.

Significant results (*p* < 0.05) were shown in bold.

### 3.4. Associations between TyG-BMI and poor sleep patterns

In the univariable and multivariable analyses, per unit-increase of TyG-BMI significantly correlated with increased odds of the presence of poor sleep patterns and components thereof, including abnormal sleep duration, trouble sleeping, and sleep disorders. In addition, individuals with the highest quartile (Q4) of TyG-BMI were significantly more likely to have all the bad sleep measures, namely, poor sleep patterns (aOR: 2.18, 95%CI: 1.61–2.95), abnormal sleep duration (aOR: 1.41, 95%CI: 1.12–1.78), trouble sleeping (aOR: 1.76, 95%CI: 1.30–2.39), and sleep disorders (aOR: 3.11, 95%CI: 2.08–4.64) as compared to those with the lowest (Q1) TyG-BMI (Table 3).

**TABLE 3:** Associations between TyG-BMI, poor sleep patterns and its components.

	Continuous	Categorical: ref: Q1 (<202.84)		
	Per 1 unit	Q2 (≥202.84, <242.13)	Q3 (≥242.13, <287.82)	Q4 (≥287.82)
**Univariate, OR (95%CI)**				
Poor sleep patterns	**1.005 (1.004–1.007)**	**1.34 (1.02–1.77)**	**1.62 (1.21–2.17)**	**2.57 (1.95–3.39)**
Abnormal sleep duration	**1.002 (1.002–1.003)**	1.20 (0.96–1.48)	**1.32 (1.12–1.56)**	**1.52 (1.24–1.87)**
Trouble sleeping	**1.004 (1.002–1.005)**	1.26 (0.99–1.61)	1.29 (0.998–1.67)	**1.97 (1.43–2.72)**
Sleep disorders	**1.008 (1.006–1.009)**	**1.51 (1.04–2.20)**	**2.17 (1.64–2.88)**	**3.90 (2.67–5.70)**
**Multivariable, aOR (95%CI)**				
Poor sleep patterns^a^	**1.005 (1.003–1.006)**	1.24 (0.91–1.68)	**1.38 (1.02–1.86)**	**2.18 (1.61–2.95)**
Abnormal sleep duration^b^	**1.002 (0.001–1.003)**	1.21 (0.97–1.51)	**1.28 (1.07–1.54)**	**1.41 (1.12–1.78)**
Trouble sleeping^c^	**1.003 (1.001–1.005)**	1.23 (0.96–1.57)	1.24 (0.98–1.57)	**1.76 (1.30–2.39)**
Sleep disorders^d^	**1.007 (1.005–1.008)**	1.34 (0.92–1.93)	**1.77 (1.36–2.29)**	**3.11 (2.08–4.64)**

^a^Adjusted for age, race, poverty income ratio, smoking status, hypertension, and CVD history.

^b^Adjusted for age, sex, race, poverty income ratio, smoking status, excessive alcohol consumption, hypertension, and CVD history.

^c^Adjusted for age, sex, race, smoking status, hypertension, CVD, and CKD history.

^d^Adjusted for age, poverty income ratio, smoking status, excessive alcohol consumption, hypertension, and CVD history.

BMI, body mass index; CVD, cardiovascular disease; CKD, chronic kidney disease; TyG, triglyceride-glucose index.

Significant results (*p* < 0.05) were shown in bold.

### 3.5. Associations between BMI component and poor sleep patterns

The association between poor sleep patterns and its components with BMI were further checked. The results showed that BMI was significantly positively associated with increased odds of all the components of poor sleep patterns (aOR: 1.02–1.07, per 1 kg/m^2^ increase of BMI) (Table 4).

**TABLE 4:** Associations between BMI, poor sleep patterns and its components.

Per 1 unit	
**Univariate model, OR (95%CI)**	
Poor sleep patterns	**1.05 (1.03–1.07)**
Abnormal sleep duration	**1.03 (1.02–1.04)**
Trouble sleeping	**1.03 (1.01–1.05)**
Sleep disorders	**1.07 (1.06–1.09)**
**Multivariate model, aOR (95%CI)**	
Poor sleep patterns^a^	**1.04 (1.03–1.06)**
Abnormal sleep duration ^b^	**1.02 (1.01–1.03)**
Trouble sleeping^c^	**1.03 (1.01–1.04)**
Sleep disorders^d^	**1.07 (1.05–1.09)**

^a^Adjusted for age, race, poverty income ratio, smoking status, hypertension, and CVD history.

^b^Adjusted for age, sex, race, poverty income ratio, smoking status, excessive alcohol consumption, hypertension, and CVD history.

^c^ Adjusted for age, sex, race, smoking status, hypertension, CVD, and CKD history.

^d^Adjusted for age, poverty income ratio, smoking status, excessive alcohol consumption, hypertension, and CVD history.

BMI, body mass index; CVD, cardiovascular disease; CKD, chronic kidney disease; TyG, triglyceride-glucose index.

Significant results (*p* < 0.05) were shown in bold.

### 3.6. Associations between TyG, TyG-BMI, short and long sleep duration

We have performed an additional analysis to examine whether the TyG indices are associated with short sleep duration (<7 h) when the individuals with long sleep duration (>9 h) are excluded, and vice versa. After adjusting for relevant confounders, TyG-BMI in Q3 (aOR: 1.34, 95%CI: 1.12–1.60) and Q4 (aOR: 1.46, 95%CI: 1.15–1.85) were significantly associated with increased odds of short sleep duration but not long sleep duration (Table 5).

**TABLE 5:** Associations between TyG, TyG-BMI, short sleep and long sleep duration.

	TyG index^a^			TyG-BMI^b^		
	Categorical: ref: Q1 (<8.16)			Categorical: ref: Q1 (<202.84)		
	Q2 (≥8.16, <8.58)	Q3 (≥8.58, <9.03)	Q4 (≥9.03)	Q2 (≥202.84, <242.13)	Q3 (≥242.13, <287.82)	Q4 (≥287.82)
**aOR (95%CI)**						
Short sleep <7 h (vs. 7–9 h)	1.13 (0.88–1.44)	1.18 (0.96–1.44)	0.95 (0.71–1.26)	1.23 (0.99–1.53)	**1.34 (1.12–1.60)**	**1.46 (1.15–1.85)**
Long sleep >9 h (vs. 7–9 h)	1.27 (0.45–3.60)	1.24 (0.50–3.12)	0.62 (0.29–1.34)	0.86 (0.32–2.34)	0.52 (0.21–1.27)	0.81 (0.44–1.50)

^a^Adjusted for age, race, BMI, poverty income ratio, smoking status, hypertension, and CVD history.

^b^Adjusted for age, race, poverty income ratio, smoking status, hypertension, and CVD history.

BMI, body mass index; CVD, cardiovascular disease; CKD, chronic kidney disease; TyG, triglyceride-glucose index.

Significant results (*p* < 0.05) were shown in bold.

## 4. Discussion

This study investigated the potential associations between poor sleep patterns and the novel surrogate indicator of metabolic dysregulation and IR, TyG and related index TyG-BMI, in a non-diabetic adult population in the US. The results indicated that individuals with a high TyG index are 1.46-fold more likely to suffer from trouble sleeping than those with a low TyG index independent of BMI. However, significant associations were not found between TyG index and the other components of poor sleep patterns, i.e., abnormal sleep duration and physician-diagnosed sleep disorders. On the other hand, individuals with high TyG-BMI are 2.18-fold more likely to have poor sleep patterns; specifically, 1.76-fold more likely to have trouble sleeping, 1.41-fold more likely to have abnormal sleep duration, and 3.11-fold more likely to have sleep disorders. For abnormal sleep duration, in particular, higher TyG-BMI levels correlate with short sleep duration <7 h but not long sleep duration >9 h. However, since higher BMI alone was significantly correlated with the components of poor sleep patterns, the correlations found between TyG-BMI, short sleep duration and sleep disorder are likely explained by the connection between poor sleep and BMI *per se*.

A prior systematic review documented that inflammatory markers such as C-reactive protein (CRP) and serum amyloid A (SAA) might be the significant mediators in the causal relationship between sleep loss and glucose intolerance. Other metabolic features such as Glucagon-like peptide-1 (GLP-1) and Non esterified fatty acids (NEFA) metabolism may also be critical (27). Findings of the review suggest that adequate sleep is necessary for maintaining proper metabolic health to prevent long-term complications such as type 2 diabetes and metabolic syndrome.

A study by Krittanawong et al. reported that average sleep duration in the US were 6.9 ± 1.4 h in all ages. And among those aged 20–79, 16.3% suffered from sleep issues (12). In the present study, 15.1% subjects had a poor sleep pattern, which is similar with the previous report.

With regard to TyG values, mean TyG of the non-diabetic cohort in the present study was 8.5. A previous NHANES study has reported that TyG ranged from 6.79 to 12.55 with a mean of 8.8 among US population over 40 years old (26). Another study using NHANES data has documented that, in US adolescents, TyG index ranged between 7.35 in Non-Hispanic Black males and 8.95 in Mexican American males (28).

Triglyceride glucose is a feasible and relatively cheap marker for IR. We found that high TyG index was significantly associated with increased prevalent self-report trouble sleeping as compared to the lowest TyG quartile independent of BMI. Studies that directly evaluated the relationship between poor sleep or self-report trouble sleeping and TyG index are lacking in the literature. However, a few studies had reported an association between TyG and one of the sleep disorders, obstructive sleep apnea (OSA) (29, 30). Bikov et al. hypothesized that OSA would elevate TyG values in non-obese patients without diabetes. Therefore, they evaluated TyG in such population and eventually correlated TyG index with markers of disease severity of OSA. It was concluded that among non-diabetic, non-obese patients with OSA, regardless of age, gender, and BMI, had higher TyG values (29). Another study demonstrated that increased TyG index was independently associated with increased OSA risk (OR: 3.348; 95% CI: 1.081–10.372) (30). However, we cannot conclude OSA was the major contributor to the correlation found between self-report trouble sleeping and TyG index based on the present results, since an OSA may not necessarily present as a self-aware “trouble sleeping.”

No statistically significant association was found between short or long sleep duration and TyG. A previous study evaluated whether there is a correlation between media time and sleep duration among children and adolescents with obesity (31). As a result, short sleep duration was associated with insulin resistance and high plasma lipoprotein, suggesting insufficient sleep and excessive media exposure may contribute to greater metabolic risk in obese children.

In this study, another related index, the TyG-BMI, was assessed in addition to TyG. The results showed that TyG-BMI correlated with not only trouble sleeping, but also short sleep duration and sleep disorders. A previous Korean study compared TyG-BMI to other TyG-related indices including TyG, TyG-waist circumference (TyG-WC), and TyG-waist-to-height ratio (TyG-WHtR), and concluded that TyG-BMI was superior to other indices in predicting IR [the areas under the receiver operating characteristic curve (ROC) curve = 0.748]. However, the authors also mentioned that the superiority of such indices is not conclusive, and further comparisons between these TyG related markers are required (25). Besides, high BMI, sleep disorders, as well as sleep quality and quantity are largely interwined, as widely documented in the literature (32, 33). In this study, we have further checked the correlations between poor sleep patterns and BMI alone, and the results showed that increased BMI is significantly associated with all the components of poor sleep patterns. In addition, the odds of elevated TyG-BMI seemed no greater than that of elevated BMI on poor sleep patterns. As such, it is reasonable for us to postulate that the connection observed between poor sleep patterns and TyG-BMI are probably best explained by the association between poor sleep and BMI *per se*.

Several other reports investigated associations between serum lipids level (e.g., cholesterols, lipoproteins, triglycerides) and shorter sleep duration. Examination of the relationship between sleep quality, sleep duration, glycemic control, and blood lipid composition among patients with diabetes in an Iranian population showed that patients with sleep disorders had significantly higher fasting blood glucose, total cholesterol, and triglycerides than those without (34), revealing that sleep conditions also interferes the blood lipid profiles. In specific, that study reported greater sleep disturbance is positively correlated with higher total cholesterol (β = 0.1) and triglycerides (β = 0.02). Other studies reported that shorter and longer sleep durations were associated with abnormal serum lipid profiles in men and women. Specifically, short sleep duration was associated with low HDL cholesterol/high triglyceride (35, 36).

As one of the major strengths of the present analysis, data from NHANES, drawing from a large and diverse sample of participants from the population of the US, are comprehensive and nationally representative, therefore, the findings are likely generalizable to the overall US population. The present study has several limitations. Firstly, it is of cross-sectional design, causal inference regarding the independent association found between TyG index and self-reported trouble sleeping could not be inferred under the cross-sectional design of the analysis. Secondly, certain foods and drinks might affect sleep quality (37), whereas such data were not included. Thirdly, other potential risk factors not recorded by NHANES might exist, hindering the reliability of the present analysis. Lastly, we used interviews and questionnaires to assess poor sleep patterns and their components instead of objective tests such as sleep studies and actigraphy. Thus, inaccurate reporting or recall bias may confound the study results. The questionnaire we used to identify a subject with sleep disorders is unspecific, which could include a spectrum of varied sleep disorders with different implications on metabolic health.

## 5. Conclusion

As a novel surrogate for IR, high TyG index is significantly associated with self-reported trouble sleeping in non-diabetic US adults, independent of BMI. The findings draw attention to the potential for metabolic changes in trouble sleepers, advising clinicians and healthcare providers to take appropriate, prompt actions in the prevention of diabetes in light of sleep conditions. Future studies should build upon this preliminary work and examine these associations longitudinally and through treatment trials.

## Data availability statement

The original contributions presented in this study are included in the article/supplementary material, further inquiries can be directed to the corresponding author.

## Ethics statement

The NCHS Research Ethics Review Board reviewed and approved the NHANES program, and all survey participants provided signed informed consent. Therefore, no further ethical approval or informed consent were required to perform the analysis of secondary data reported in this manuscript. Please check the NHANES website for NCHS Research Ethics Review Board Approval (https://www.cdc.gov/nchs/nhanes/irba98.htm).

## Author contributions

C-FL: acquisition of data, analysis and interpretation of data, drafting of the manuscript, guarantor of integrity of the entire study, and statistical analysis. L-WC: conception and design, critical revision of the manuscript, statistical analysis, and guarantor of integrity of the entire study. Both authors contributed to the article and approved the submitted version.

## Footnotes

1
http://www.cdc.gov/nchs/nhanes/

2
https://www.cdc.gov/nchs/nhanes/irba98.htm

## References

[1] Lebovitz HE. Insulin resistance: definition and consequences. *Exp Clin Endocrinol Diabetes.* (2001) 109 (Suppl. 2):S135–48. 10.1055/s-2001-18576 11460565

[2] Lee SH Park SY Choi CS. Insulin resistance: from mechanisms to therapeutic strategies. *Diabetes Metab J.* (2022) 46:15–37. 10.4093/dmj.2021.0280 34965646 PMC8831809

[3] Yaribeygi H Farrokhi FR Butler AE Sahebkar A. Insulin resistance: review of the underlying molecular mechanisms. *J Cell Physiol.* (2019) 234:8152–61. 10.1002/jcp.27603 30317615

[4] Hong S Han K Park CY. The insulin resistance by triglyceride glucose index and risk for dementia: population-based study. *Alzheimers Res Ther.* (2021) 13:9. 10.1186/s13195-020-00758-4 33402193 PMC7786939

[5] Zhu B Wang J Chen K Yan W Wang A Wang W A high triglyceride glucose index is more closely associated with hypertension than lipid or glycemic parameters in elderly individuals: a cross-sectional survey from the Reaction Study. *Cardiovasc Diabetol.* (2020) 19:112. 10.1186/s12933-020-01077-6 32664945 PMC7362407

[6] Ma M Liu H Yu J He S Li P Ma C Triglyceride is independently correlated with insulin resistance and islet beta cell function: a study in population with different glucose and lipid metabolism states. *Lipids Health Dis.* (2020) 19:121. 10.1186/s12944-020-01303-w 32487177 PMC7268278

[7] Prentki M Matschinsky FM Madiraju SR. Metabolic signaling in fuel-induced insulin secretion. *Cell Metab.* (2013) 18:162–85. 10.1016/j.cmet.2013.05.018 23791483

[8] Kang B Yang Y Lee EY Yang HK Kim HS Lim SY Triglycerides/glucose index is a useful surrogate marker of insulin resistance among adolescents. *Int J Obes.* (2017) 41:789–92. 10.1038/ijo.2017.14 28104918

[9] da Silva A Caldas APS Hermsdorff HHM Bersch-Ferreira AC Torreglosa CR Weber B Triglyceride-glucose index is associated with symptomatic coronary artery disease in patients in secondary care. *Cardiovasc Diabetol.* (2019) 18:89. 10.1186/s12933-019-0893-2 31296225 PMC6625050

[10] Shi W Xing L Jing L Tian Y Yan H Sun Q Value of triglyceride-glucose index for the estimation of ischemic stroke risk: insights from a general population. *Nutr Metab Cardiovasc Dis.* (2020) 30:245–53. 10.1016/j.numecd.2019.09.015 31744716

[11] Bruce ES Lunt L McDonagh JE. Sleep in adolescents and young adults. *Clin Med.* (2017) 17:424–8. 10.7861/clinmedicine.17-5-424 28974591 PMC6301929

[12] Krittanawong C Kumar A Wang Z Jneid H Baber U Mehran R Sleep duration and cardiovascular health in a representative community population (from NHANES, 2005 to 2016). *Am J Cardiol.* (2020) 127:149–55. 10.1016/j.amjcard.2020.04.012 32466847

[13] Adir Y Humbert M Chaouat A. Sleep-related breathing disorders and pulmonary hypertension. *Eur Respir J.* (2021) 57:2002258. 10.1183/13993003.02258-2020 32747397

[14] Hepburn M Bollu PC French B Sahota P. Sleep medicine: stroke and sleep. *Mo Med.* (2018) 115:527–32. 30643347 PMC6312177

[15] Huang T Mariani S Redline S. Sleep irregularity and risk of cardiovascular events: the multi-ethnic study of atherosclerosis. *J Am Coll Cardiol.* (2020) 75:991–9. 10.1016/j.jacc.2019.12.054 32138974 PMC7237955

[16] Knowlden AP Higginbotham JC Grandner MA Allegrante JP. Modeling risk factors for sleep- and adiposity-related cardiometabolic disease: protocol for the short sleep undermines cardiometabolic health (SLUMBRx) observational study. *JMIR Res Protoc.* (2021) 10:e27139. 10.2196/27139 33687340 PMC7988396

[17] Saeed S Romarheim A Solheim E Bjorvatn B Lehmann S. Cardiovascular remodeling in obstructive sleep apnea: focus on arterial stiffness, left ventricular geometry and atrial fibrillation. *Expert Rev Cardiovasc Ther.* (2022) 20:455–64. 10.1080/14779072.2022.2081547 35673889

[18] Antza C Kostopoulos G Mostafa S Nirantharakumar K Tahrani A. The links between sleep duration, obesity and type 2 diabetes mellitus. *J Endocrinol.* (2021) 252:125–41. 10.1530/JOE-21-0155 34779405 PMC8679843

[19] Damanti S Bourron O Doulazmi M Mandengue Sosso AL Nguyen-Michel VH Mariani J Relationship between sleep parameters, insulin resistance and age-adjusted insulin like growth factor-1 score in non diabetic older patients. *PLoS One.* (2017) 12:e0174876. 10.1371/journal.pone.0174876 28384333 PMC5383056

[20] Zipf G Chiappa M Porter KS Ostchega Y Lewis BG Dostal J. National health and nutrition examination survey: plan and operations, 1999-2010. *Vital Health Stat.* (2013) 1:1–37. 25078429

[21] American Diabetes Association. Diagnosis and classification of diabetes mellitus. *Diabetes Care.* (2013) 36 (Suppl. 1):S67–74. 10.2337/dc13-S067 23264425 PMC3537273

[22] Chaput JP Dutil C Sampasa-Kanyinga H. Sleeping hours: what is the ideal number and how does age impact this?. *Nat Sci Sleep.* (2018) 10:421–30. 10.2147/NSS.S163071 30568521 PMC6267703

[23] Li C Shang S. Relationship between sleep and hypertension: findings from the NHANES (2007-2014). *Int J Environ Res Public Health.* (2021) 18:7867. 10.3390/ijerph18157867 PMC8345503 34360157

[24] Guerrero-Romero F Simental-Mendia LE Gonzalez-Ortiz M Martinez-Abundis E Ramos-Zavala MG Hernandez-Gonzalez SO The product of triglycerides and glucose, a simple measure of insulin sensitivity. Comparison with the euglycemic-hyperinsulinemic clamp. *J Clin Endocrinol Metab.* (2010) 95:3347–51. 10.1210/jc.2010-0288 20484475

[25] Lim J Kim J Koo SH Kwon GC. Comparison of triglyceride glucose index, and related parameters to predict insulin resistance in Korean adults: an analysis of the 2007-2010 Korean national health and nutrition examination survey. *PLoS One.* (2019) 14:e0212963. 10.1371/journal.pone.0212963 30845237 PMC6405083

[26] Wu TD Fawzy A Brigham E McCormack MC Rosas I Villareal DT Association of triglyceride-glucose index and lung health: a population-based study. *Chest.* (2021) 160:1026–34. 10.1016/j.chest.2021.03.056 33839084 PMC8449007

[27] Singh T Ahmed TH Mohamed N Elhaj MS Mohammed Z Paulsingh CN Does insufficient sleep increase the risk of developing insulin resistance: a systematic review. *Cureus.* (2022) 14:e23501. 10.7759/cureus.23501 35494895 PMC9036496

[28] Moon S Park JS Ahn Y. The Cut-off values of triglycerides and glucose index for metabolic syndrome in American and Korean adolescents. *J Korean Med Sci.* (2017) 32:427–33. 10.3346/jkms.2017.32.3.427 28145645 PMC5290101

[29] Bikov A Frent SM Meszaros M Kunos L Mathioudakis AG Negru AG Triglyceride-glucose index in non-diabetic, non-obese patients with obstructive sleep apnoea. *J Clin Med.* (2021) 10:1932. 10.3390/jcm10091932 33947164 PMC8125770

[30] Kang HH Kim SW Lee SH. Association between triglyceride glucose index and obstructive sleep apnea risk in Korean adults: a cross-sectional cohort study. *Lipids Health Dis.* (2020) 19:182. 10.1186/s12944-020-01358-9 32771021 PMC7414547

[31] Sayin FK Buyukinan M. Sleep duration and media time have a major impact on insulin resistance and metabolic risk factors in obese children and adolescents. *Child Obes.* (2016) 12:272–8. 10.1089/chi.2015.0126 26978730

[32] Ogilvie RP Patel SR. The epidemiology of sleep and obesity. *Sleep Health.* (2017) 3:383–8. 10.1016/j.sleh.2017.07.013 28923198 PMC5714285

[33] St-Onge MP. Sleep-obesity relation: underlying mechanisms and consequences for treatment. *Obes Rev.* (2017) 18 (Suppl. 1):34–9. 10.1111/obr.12499 28164452 PMC13098705

[34] Barikani A Javadi M Rafiei S. Sleep quality and blood lipid composition among patients with diabetes. *Int J Endocrinol Metab.* (2019) 17:e81062. 10.5812/ijem.81062 31497039 PMC6678070

[35] Du J Chen Y Zhou N Song Y Wang W Hong X. Associations between self-reported sleep duration and abnormal serum lipids in eastern China: a population-based cross-sectional survey. *Sleep Med.* (2022) 95:1–8. 10.1016/j.sleep.2022.04.004 35533627

[36] Smiley A King D Harezlak J Dinh P Bidulescu A. The association between sleep duration and lipid profiles: the NHANES 2013-2014. *J Diabetes Metab Disord.* (2019) 18:315–22. 10.1007/s40200-019-00415-0 31890656 PMC6914752

[37] Binks H E Vincent G Gupta C Irwin C Khalesi S. Effects of diet on sleep: a narrative review. *Nutrients.* (2020) 12:936. 10.3390/nu12040936 32230944 PMC7230229

